# Perioperative Morbidities in Distal Radius Fractures Treated Using Locking Plates in the Super-Elderly Population: A Retrospective Study

**DOI:** 10.1016/j.jhsg.2022.11.004

**Published:** 2022-12-22

**Authors:** Bérénice Moutinot, Ivana Sojevic, Cindy Bouvet, Olivier Mares, Manon Vouga, Jean-Yves Beaulieu

**Affiliations:** ∗ Department of Orthopaedic Surgery, Geneva University Hospitals, Geneva, Switzerland; † Department of Orthopaedic Surgery, Nîmes University Hospital, Nîmes, France; ‡ Department of Obstetrics and Gynecology, Lausanne University Hospital, Lausanne, Switzerland

**Keywords:** Complications, Distal radius fracture, Elderly, Rehabilitation

## Abstract

**Purpose:**

Currently, there is no consensus on the treatment of distal radius fractures in the super-elderly population. The aim of this study was to evaluate the perioperative morbidities and the need for rehabilitation care after a distal radius fracture treated with locking plates among patients aged 85 years or older.

**Methods:**

A retrospective study was conducted in all patients aged 85 years or older who underwent open surgical treatment using a locking plate for an isolated distal radius fracture from January 2013 to December 2018 at a level 1 trauma center. The occurrence of minor complications (tendinopathy, neuropathy, carpal tunnel syndrome, and infection), major complications (complex regional pain syndrome, nonunion, loss of reduction, intra-articular screw, and hardware failure), and the need for revision surgery were recorded. The need and timing of rehabilitation were also documented. A nested case-control study was performed to evaluate predictive factors associated with the need for inpatient rehabilitation.

**Results:**

The majority of fractures were AO type A, numbering 88 (55.7%), followed by 64 type C (40.5%), and then 6 type B (3.8%). The overall complication rate among the 158 included patients was 17% (n = 26), with 12 (7.6%) having minor complications and 14 (8.9%) having major complications. Inpatient rehabilitation was required for one-third of the patients (n = 59), and 11 (7%) were definitively discharged to a nursing home. The place of residence before the fracture, American Society of Anesthesiologist score, and the type of anesthesia were associated with a need for inpatient rehabilitation.

**Conclusions:**

Overall, this study suggests that perioperative morbidity of distal radius fractures treated using a locking plate is acceptable even in the super-elderly population. Nevertheless, given the frequent requirement for rehabilitation, the impact of age cannot be ignored.

**Type of Study/level of evidence:**

Therapeutic IV.

The management of distal radius fractures in older adults remains a controversial subject. Indeed, with the advent of volar locking plates in the 2000s, surgical management became popular among older adult patients (aged >60 years), and there was a significant increase in the number of procedures performed each year.^1^ Fractures of the distal end of the radius are the second most common fracture after hip fractures among the elderly population, representing 17% of all fractures.^2^, ^3^, ^4^, ^5^, ^6^, ^7^ However, age is not the only determinant of recovery capacity and the risk of surgical complications; other factors may influence the postoperative outcome.

The aging of the population, combined with the progress in medicine, has contributed to the emergence of a new class of older adults called “super seniors.”^8^ This refers to patients aged 85 years and older. The health condition of individuals in this age group is in a delicate balance. The slightest loss of autonomy, even partial, such as prolonged immobilization with a cast, could have negative repercussions on their future.

The long-term functional outcomes between operative and conservative management of distal radius fractures are still debated.^9^, ^10^, ^11^, ^12^ Living at home is sometimes based on a fragile state. The surgical option in this age group could be justified by the shorter immobilization time and, therefore, the better chance of regaining autonomy. It remains unclear whether the complication rate after distal radius plating is acceptable in this fragile population. The aim of this study was to determine the surgical morbidity of distal radius fractures treated by using locking plates among patients aged 85 years and older and to determine the need for rehabilitation in this population in the short and medium term. We hypothesized that perioperative morbidity after distal radius fracture in geriatric patients is low despite advanced age.

## Materials and Methods

### Study overview

This was a retrospective cohort study. Local audit approval was obtained (Swiss ethic ID 2020-02594) and written informed consent was obtained from a legally authorized representative(s) for anonymized patient information to be published in this article. Patients who were aged 85 years or older and were operated on in a university hospital for an isolated distal radius fracture with a locking plate from 2013 to 2018 were included. The hospital concerned provides both acute and rehabilitation care. The patients were selected for this study by performing a search of the hospital’s computer database.

For all patients, we collected the following outcomes: sex, age, comorbidities, American Society of Anesthesiologist (ASA) category, the type of anesthesia (axillary block or general anesthesia—general anesthesia was considered only when regional anesthesia failed or when the patient was not compliant), body mass index, the type of fracture (according to the AO classification simplified as the following: [1] A, extra-articular; [2] B, partially intra-articular; and [3] C intra-articular), the place of residence before and after the fracture (nursing or private home), hospitalization time (acute and rehabilitation unit), minor complications (tendinopathy, neuropathy, carpal tunnel syndrome, and infection), major complications (complex regional pain syndrome, nonunion, loss of reduction, intra-articular screw, and hardware failure), and the need for revision surgery (new fixation, carpal tunnel release, early removal of fracture fixation device, superficial debridement, surgical site lavage, tendon transfer, and ulnar head resection). Complex regional pain syndrome was considered a major complication because it can have a devastating impact on the patient’s independence.^13^, ^14^, ^15^ Mental illness, as a comorbidity, included a broad range of disabilities, from mild cognitive impairment mentioned in the medical record to severe neurological pathology (eg, Alzheimer disease, frontotemporal dementia).

The overall rate of complications was defined as the share of patients with either a major or minor complication, or both. The major complication rate was defined as the rate of any major complication independent of the presence of associated minor complications. The minor complication rate was defined as the rate of minor complications excluding patients with major complications. Early removal was defined as removal of the fixation hardware within 1 year of the initial surgery; this was done in cases of discomfort or symptoms of flexor tenosynovitis.

Moreover, the standard postoperative immobilization protocol was 2 weeks of strict wearing of a short-arm cast, followed by 4 weeks of an orthosis and free mobilization.

All patients were hospitalized for at least 1 night for postoperative pain control and the prevention of edema. If patients were not sufficiently independent to care for themselves at home with an immobilized wrist the next day, they were then offered a transfer to an inpatient rehabilitation ward. The timing of the transfer from acute care units to rehabilitation depended on the availability of rehabilitation places at the time of the request.

### Statistical methods

The absolute risks and their 95% confidence intervals (95% CIs) were estimated by proportions using the exact binomial score.

To evaluate predictive factors associated with the need for postoperative rehabilitation, we performed a nested case-control study comparing patients who required postoperative rehabilitation—who were considered as cases—to patients who did not require rehabilitation—who were considered as controls. The effects of suspected clinical risk factors were tested (ie, sex, age of ≥90 years, dichotomized as <90 or ≥90 years), potential comorbidities summarized using the ASA category (dichotomized as ASA 1–2 vs ASA 3–4), the prefracture place of residence (nursing home vs private home or hospital), the presence of complications (minor, major, or the need for revision surgery), and type of anesthesia (dichotomized as general anesthesia vs axillary block). We performed a multivariate logistic regression analysis to estimate odds ratios (ORs) with 95% CIs of postoperative need for rehabilitation adjusted for suspected clinical risk factors and the presence of potential postoperative complications.

## Results

A total of 158 patients aged 85 years or older who underwent distal radius fixation using a locking plate between 2013 and 2018 were included. There was a female predominance, and the mean age was 89 years (min 85, max 102). Most patients were still living at home (n = 124) at the time of the fracture; 22 (14%) came from a nursing home, and 12 (7.8%) were already hospitalized for other reasons and fell on their wrist during their stay. More than half the patients (n = 82) were classified as ASA 2. Most patients (n = 134) were operated on with an axillary block, and all patients had a volar locking plate, except 2 who had a dorsal locking plate (Table 1).

**Table 1:** Demographic Data

	Patients (N = 158), No. (%)	
Age (y)		
Median (min–max)	89	(85–102)
85–90	103	(65.19)
>90	55	(34.81)
Sex		
Male	11	(6.96)
Female	147	(93.04)
Comorbidities		
Kidney failure	27	(17.09)
Alcoholism	4	(2.53)
Cardiovascular disease∗	78	(49.37)
Diabetes	8	(5.06)
High blood pressure	107	(67.72)
Smoking	2	(1.27)
Osteoporosis	62	(39.24)
Mental illness	46	(29.11)
Active cancer	13	(8.23)
BMI		
Median (IQR)	23	(20.8–25.99)
<18.5	12	(7.59)
18.5–25	89	(56.33)
>25	57	(36.08)
ASA score		
1	3	(1.9)
2	82	(51.9)
3	72	(45.6)
4	1	(0.6)
Type of anesthesia		
Axillary block	134	(84.8)
General anesthesia	24	(15.2)
Residence at the time of fracture		
Private home	124	(78.5)
Nursing home	22	(13.9)
Hospital	12	(7.8)
AO classification of fracture		
A	88	(55.7)
B	6	(3.8)
C	64	(40.5)
Follow-up (mos)		
Median (IQR)	3.5	(2.5–6)

Acute carpal tunnel release 16 (10)

BMI: Body mass index, IQR: Interquartile range.

∗ Includes any myocardial infarction, cardiac failure, cardiac arrhythmia, and stroke or peripheral arterial disease.

The overall complication rate was 17% (n = 26), with a 7.6% rate of minor complications (n = 12) and an 8.9% rate of major complications (n = 14) (Table 2). Four patients had both major and minor complications.

**Table 2:** Complications, Revision Surgeries, and Destination After Discharge

	N = 158, No. (%)		95% CI
Complications			
Any complication (total)	26	(16.5)	11.0–23.2
Minor complications only	12	(7.6)	4.0–12.9
Major complications	14	(8.9)	4.9–14.4
Any minor complication	16	(10.1)	5.9–15.9
Carpal tunnel syndrome	4	(2.5)	0.7–6.4
Dysesthesia	3	(1.9)	0.4–5.4
Infection	0	(0)	0–2.3
Tendinopathy	9	(5.7)	2.6–10.5
Any major complication	14	(8.9)	4.9–14.4
Complex regional pain syndrome	1	(0.6)	0.0–3.5
Nonunion	2	(1.3)	0.2–4.5
Malreduction	0	(0)	0.0–2.3
Reduction loss	6	(3.8)	1.4–8.1
Intra-articular screw	4	(2.5)	0.7–6.4
Hardware failure	5	(3.2)	1.0–7.2
Revision surgery			
Any revision surgery	12	(7.6)	4.0–12.9
Refixation of fracture	3	(1.9)	0.4–5.4
Secondary carpal tunnel release	2	(1.3)	0.2–4.5
Early hardware removal	8	(5.1)	2.2–9.7
Superficial debridement	0	(0)	0.0–2.3
Lavage	0	(0)	0.0–2.3
Tendon transfer	1	(0.6)	0.0–3.5
Ulnar head resection (Darrach)	1	(0.6)	0.0–3.5
Destination after discharge			
Home	98	(62.0)	54.0–69.6
Rehabilitation facility	59	(37.3)	29.8–45.4
Died	1	(0.6)	0.0–3.5

CI, confidence interval.

Tendinopathy was the leading minor complication in 9 (5.7%) patients, followed by carpal tunnel syndrome in 4 (2.5%). Sixteen (10%) patients had primary carpal tunnel release at arrival for symptoms of acute median nerve compression.

Among the patients with minor complications (n = 12), only 2 needed revision surgery (carpal tunnel release in both).

The most common major complication was the loss of reduction (3.8%, 6 patients). One was due to another fall on the operated wrist 1 month after the initial surgery, 1 was not compliant with cast immobilization, and the rest were due to spontaneous articular collapse. Among patients who had a major complication (n = 14), 8 needed surgical revision. A subgroup analysis found no difference in major and minor complications between extra-articular (type A) and intra-articular (type B and C) fractures (Table 3).

**Table 3:** Severity of Complications by Fracture Type

	Type A Fractures			Type B/C Fractures		
	N = 88, No.	(%)	95% CI	N = 70, No.	(%)	95% CI
Major complications∗	7	(7.95)	3.5–15.7	7	(10)	4.1–19.5
Minor complications†	6	(6.8)	2.5–14.3	6	(8.6)	3.2–17.7

∗ Complex regional pain syndrome, nonunion, loss of reduction, intra-articular screw, and hardware failure.

† Tendinopathy, neuropathy, carpal tunnel syndrome, and infection.

The revision surgery rate was 7.6% (n = 12) and the most common intervention was early hardware removal in 8 (5.1%) patients. The next most common revision procedures were carpal tunnel release and refixation of the fracture, performed in 3 (1.9%) and 2 (1.3%) patients, respectively).

The mean length of hospital stay in the acute care unit was 7.7 days. One-third of the patients (n = 59) needed a rehabilitation stay, whereas two-thirds (n = 98) returned directly to their previous residence (private or nursing homes). The median length of stay in the rehabilitation facility was 37 days (interquartile range, 13.5–49). Among the patients who resided at home before the fracture, 11 (7%) never returned home and needed to be transferred to a nursing home after the operation.

In the nested case-control analysis, having a nursing home as the previous place of residence (crude OR, 0.1; 95% CI, 0.0–0.4) was associated with a significantly lower risk of rehabilitation transfer.

In a multivariate analysis adjusting for risk factors of rehabilitation need, a higher ASA score (aOR, 2.3; 95% CI, 1.1–4.7), and the use of general anesthesia (aOR, 3.2; 95% CI, 1.1–9.5) were also significantly associated. Age, sex, and having a complication (minor or major or both) or revision surgery did not appear to influence the need for rehabilitation (Table 4).

**Table 4:** Predictive Factors for Rehabilitation

	No Rehabilitation Required			Rehabilitation Required			OR (95% CI)	*P* Value	aOR∗ (95% CI)	*P* Value
	N = 99, No.	(%)	95% CI	N = 59, No.	(%)	95% CI				
Sex										
Female	92	(92.9)	86.0–97.1	55	(93.2)	83.5–98.1	1.0 (0.25–5.1)	.9446	1.1 (0.3–4.5)	.888
Age (y)										
>90	30	(30.3)	21.5–40.4	25	(42.4)	29.6–55.9	1.7 (0.8–3.5)	.1234	1.6 (0.8–3.5)	.202
Previous residence										
Nursing home	21	(21.2)	13.6–30.6	1	(1.7)	0.0–9.1	0.1 (0.0–0.4)	.0006	0.0 (0.0–0.3)	.002
ASA category										
3–4	39	(39.4)	29.7–49.7	34	(57.6)	44.1–70.4	2.1 (1.0–4.3)	.0262	2.3 (1.1–4.7)	.025
Major complication	8	(8.1)	3.6–15.3	6	(10.2)	3.8–20.8	1.3 (0.3–4.5)	.6550	1.1 (0.2–5.2)	.878
Minor complication	8	(8.1)	3.6–15.3	4	(6.8)	1.9–16.5	0.8 (0.2–3.3)	.7652	0.5 (0.1–2.2)	.386
Revision surgery	8	(8.1)	3.6–15.3	4	(6.8)	1.9–16.5	0.8 (0.2–3.3)	.7652	0.8 (0.1–4.3)	.796
Type of anesthesia										
General anesthesia	12	(12.1)	6.4–20.2	12	(20.3)	11.0–32.8	1.9 (0.7–4.9)	.1639	3.2 (1.1–9.5)	.039

CI, Confidence interval.

∗ Adjusted for sex (female or not), age (age >90 years or ≤90), previous residence (already in a nursing home vs at home or already hospitalized), ASA category 3–4 versus 1–2, major complications (including those with minor complications) versus no complication, minor complications only, and type of anesthesia (general anesthesia or not).

## Discussion

In this study, we present the largest cohort assessing super-elderly patients, with a mean age of 89 years, who had an isolated distal radius fracture treated using a locking plate. We found an overall complication rate of 17%, with 8.9% of patients having major complications and 7.6 % having minor complications. The complication rate varies widely in the literature.^16^, ^17^, ^18^, ^19^, ^20^ Our complication rate appears high compared with the 3% rate described by Heng et al,^18^ who observed patients aged over 80 years. However, their study did not include minor complications. On the contrary, another study by Lutz et al^21^ on patients aged 65 years and older, which included minor complications, found a 29% complication rate. This higher rate can be explained by the different surgical techniques used, including an external fixator, which leads to more infections. In this same study, the complication rate for the conservative treatment group was 13%, and the number of patients requiring reintervention was similar between the operated and nonoperated groups. Another study found a 14% complication rate in the conservative treatment group, which is similar to our results based on operated patients.^9^

Even if comparing the onset of complications in nonoperated patients is beyond the scope of this article, it is interesting that complication rates seem to be quite close. It is also worth mentioning that more than half of the patients included in our study had suffered an extra-articular fracture, which may have positively influenced the outcomes.

A study by Chung et al^22^ found that the most common complication in patients aged over 60 years was median nerve compression (18.5%). In our study, postoperative median nerve compression occurred in only 4 (2.5%) patients and only half of them underwent secondary carpal tunnel release. This low rate could be explained by the fact that 10% of our patients had primary carpal tunnel release during the distal radius fracture fixation surgery due to acute symptoms present upon arrival. Knowing that age is a risk factor for carpal tunnel syndrome, opening the transverse carpal concurrently with the primary fixation could be an option for super-elderly patients.

Moreover, in our study, the severity of some complications was not always proportional to the extent of treatment. Of the 14 patients with major complications (nonunion, malreduction, intra-articular screw, reduction loss, hardware displacement, and complex regional pain syndrome), 6 (43%) patients never requested reoperation because they were completely asymptomatic. In our study, only 1 patient out of 4 who had an intra-articular screw was symptomatic and had the hardware removed. It is surprising that screws protruding into the joint were so well tolerated in this elderly population. According to Clement et al, malunion does not influence the functional outcome in the super-elderly population.^23^ Previous publications also showed no link between reduction loss and poor functional outcomes.^11^^,^^23^, ^24^, ^25^ A recent article by Sagerfors et al^26^ found that, in general, patient-related outcomes in the elderly (aged >80 years) were poorer 1 year after sustaining a distal radius fracture compared with the prefracture state. However, no difference was found between conservative treatment and volar plating.

A meta-analysis of 55 studies assessing complications after volar plating in distal radius fractures in the general population (average age 57 years) found an overall complication rate of 15%: 5% major complications and 10% minor complications.^27^ These rates are very close to the complication rates found in our study despite including a super-elderly population. The difference in our rate of major complications (8.9%) could be explained by the definition of a “major complication,” which excluded carpal tunnel release, complex regional pain syndrome, and plate removal requested by the patient in that study. Furthermore, the study from Lizaur-Utrilla et al^28^ found no difference in the complication rate between younger (mean age 42 years) and older (mean age 68 years) patients. These elements seem to reinforce the fact that age should not be an obstacle to performing surgical fracture fixation in the wrist.

Ten percent of all women will sustain a distal radius fracture after 65 years of age.^2^ This female predominance found in literature matches our results.^18^^,^^29^, ^30^, ^31^ Moreover, distal radius fractures are often the result of a low-energy fall on an outstretched wrist.^3^^,^^32^ Older independent patients in good cognitive health seem to be at higher risk of sustaining a distal radius fracture as they still have the reflex to put their arm forward when they fall.^33^^,^^34^ Most of the patients in our study were living independently at home when their fracture occurred, although 29% had a known mental disorder. According to Plassman et al,^35^ the prevalence of dementia in the elderly population is approximately 24% for people aged between 80–89 years. This rate also matches our result.

Among the 158 patients included in this study, one-third (37%) needed a rehabilitation stay. Age and the place of residence before the fracture influenced the need for rehabilitation. Indeed, if patients were already in a nursing home, they then returned to the nursing home without going to a rehabilitation facility because the nursing home provides the care they need.

Lübekke et al^31^ observed that if a patient with any upper arm fracture resided at home, then their chances of needing rehabilitation were higher. Our study found that 7% of patients living at home went into a nursing home permanently after sustaining a distal radius fracture, which demonstrates the frailty of this particular population.

In addition, the follow-up of our patients was relatively short; this can influence the rate of complications, such as tendon ruptures, which can occur years after the initial fracture fixation.^20^^,^^36^ In practice, most patients who had recovered well 3 months after surgery, as evidenced by a healed fracture on radiographs, refused to continue medical monitoring. These patients were told to return if any problems reappeared, but none did.

Additionally, we must make allowances for selection bias as the decision to operate on a patient was made on a case-by-case basis by the surgeon, who may have preferred to operate on a fit, super-elderly patient rather than one with multiple comorbidities. The low proportion of patients with an ASA 4 score in our study seems to support this fact. Moreover, comparing the complication rate with other studies is difficult, as patient selection varies. It would have been useful to have a comparator group whose fracture was treated conservatively.

In conclusion, distal radius plating is becoming increasingly popular among super-elderly patients who are often fitter than younger patients. Super-elderly patients have a 7% risk of being placed in a nursing home after sustaining a distal radius fracture treated using a locking plate. Age should not be a critical factor influencing treatment, because the overall perioperative morbidity is acceptable even in the super-senior population and rare complications do not always correlate with symptoms.
